# Economic Attitudes, Social Attitudes and Their Psychological Underpinnings – A Study of the Finnish Political Elite

**DOI:** 10.3389/fpsyg.2019.00602

**Published:** 2019-03-19

**Authors:** Jan-Erik Lönnqvist, Matias Kivikangas

**Affiliations:** ^1^Swedish School of Social Science, University of Helsinki, Helsinki, Finland; ^2^KU Leuven, Leuven, Belgium

**Keywords:** political attitudes, political elite, left-right dimension elections, liberal-conservative, authoritarianism, social dominance orientation, dual process models, political ideology

## Abstract

We investigated the relation between economic and social attitudes and the psychological underpinnings of these attitudes in candidates (*N* = 9515) in the Finnish 2017 municipal elections. In this politically elite sample, right-wing economic attitudes and social conservatism were positively correlated (*r* = 0.41), and this correlation was predominantly driven by those on the economic left being socially liberal, and vice versa. In terms of underlying psychological processes, consistent with dual process models of political ideology, the anti-egalitarian aspect of social dominance orientation was more strongly associated with right-wing economic attitudes, and the conventionalism and aggression aspects of right-wing authoritarianism with social conservatism. Our results show that even in a non-United States context in which the masses organize their political attitudes on two independent dimensions, these dimensions are moderately aligned among certain parts of the political elite, and that the political attitudes of the political elite can be traced to underlying psychological motivations. We argue that equality concerns could play a role in explaining why the left-right and liberal-conservative dimensions are more strongly aligned among those on the left and those more liberal.

## Introduction

Although political psychology is often defined as an interdisciplinary field in which psychological concepts and methods are used to test theories about elite and mass political behavior, most of the empirical research has focused on mass publics. Moreover, much of this research has been conducted in a two-party system – the United States. From this literature, we know that (1) economic and social attitudes are more strongly aligned among the highly educated and politically engaged, (2) right-wing people tend to show more heterogeneity in their social attitudes, and (3) economic and social attitudes have distinct psychological underpinnings, an idea often referred to as the dual process model of ideology. The purpose of the present research was to extend upon this literature by investigating to what extent similar patterns can be found in the political elite in a typical Western European multi-party system.

More specifically, we investigated the relation between economic and social attitudes and the psychological underpinnings of these attitudes among the almost 10,000 candidates in the Finnish 2017 municipal elections who had responded to the two largest voting advice applications (VAAs) hosted by the largest national newspaper, Helsingin Sanomat, and by the Finnish National Broadcasting company, YLE. The former application included measures of economic and social attitudes and the latter included items adapted from measures of right-wing authoritarianism and social dominance orientation, two of the currently most researched conceptualizations of the psychological underpinnings of political attitudes.

### The Economic and Social Dimensions of Political Attitudes

Prior research has provided conflicting accounts regarding the extent to which economic and social political attitudes tend to be aligned on one dimension. Although the language and rhetoric of the political elite – politicians, social scientists, philosophers, and the media – often operationalizes political attitudes as a unidimensional continuum, ranging from left to right, or liberal to conservative, research on public opinion has since the 1960s suggested that this may be a poor description of the political attitudes of the masses (Converse, 1964). A considerable body of work now demonstrates that political attitudes are often multifaceted, better described by two dimensions–an economic/fiscal dimension and a social/cultural dimension (Lane, 1962; Conover and Feldman, 1981; Duckitt, 2001; Weber and Federico, 2007).

Regarding the economic dimension of attitudes, right-wing (as opposed to left-wing) economic policies focus on a large role for free markets and individual action, rejection or retrenchment of the welfare state, whereby the state’s role is limited to the provision of public goods and the correction of market failures, and general economic orthodoxy, revolving around the ideas of limited public finance, balanced government budgets, and strong property rights (e.g., Budge et al., 2001; Klingemann et al., 2006). By contrast, left-wing economic ideas build on the necessity of regulating the free market. Such ideas include the expansion of the welfare state and education (Bobbio, 1996), claims for economic planning and market regulation, and also more radical ideas about the nationalization of enterprises and government control of the economy (Budge et al., 2001; Klingemann et al., 2006; Jahn, 2011). In the Finnish context, differences between the left and the right often revolve around taxes, public welfare expenditure, privatization, and deregulation (Karvonen, 2014).

Social conservatism (as opposed to liberalism) can broadly speaking be defined as “resistance to change and the tendency to prefer safe, traditional and conventional forms of institutions and behavior” (Wilson, 1973, p. 4). A central tenet of social conservatism is that for social harmony, a certain amount of consistency needs to be maintained in the social fabric. Social conservatism emphasizes the importance of preserving ties that bind people together, such as family, religion and customs, making traditional morality and a national way of life central for social conservatism (Budge et al., 2001; Klingemann et al., 2006). In the Finnish context, typical dividing issues between social conservatives and liberals are immigration, same-sex marriage, same-sex adoption, the role of religion and traditional morality in politics, and patriotism (Karvonen, 2014).

### Determinants of the Alignment Between Economic and Social Attitudes

The extent to which the economic and social dimensions of political attitudes are aligned has been shown to depend on both cultural and individual-level characteristics (Jost et al., 2009). The two dimensions of political attitudes have been historically intertwined: As Western societies have moved incrementally toward greater equality, progress has meant increased egalitarianism, whereas resistance to change has generally been associated with maintenance of traditional, more hierarchical forms of social organization. However, in the last decades, the organization of political attitudes has been argued to have become increasingly two-dimensional (Bornschier, 2010). Indeed, using survey data from 99 nations, a recent study found that the alignment of right-wing economic attitudes (acceptance of inequality) with social conservatism (adherence to tradition) is globally uncommon (Malka et al., 2017). The unidimensional continuum that tends to organize the language and rhetoric of the political elite is more likely to be found within modern and developed nations, and even there, primarily in two-party or two-bloc systems, such as the United States or the United Kingdom (Malka et al., 2017). Research employing the nationally representative data sets of the World Values Survey shows that also in Finland, as well as many other Western European countries, the correlation between the economic and social dimensions of political attitudes is virtually zero among the mass public (Malka et al., 2017). However, this may not necessarily be the case among the political elite.

At least in some contexts, alignment between the economic and social dimensions is conditional on a person’s level of political engagement. In the United States, those who are highly educated (Sidanius and Duffy, 1988) and highly politically engaged (Sidanius and Duffy, 1988; Federico and Schneider, 2007) are the most likely to organize their economic and social attitudes along one dimension, whereas those low in political engagement are more likely to adopt a “mixed bag” of attitudes (for a review, see Feldman, 2013). This has generally been argued to be a consequence of the more educated and politically engaged following the elite political competition that in a two-party system such as the United States tends to occur along only one dimension (Feldman and Johnston, 2014). We will investigate whether political engagement is similarly associated with a more unidimensional structuring of political attitudes in a context such as the Finnish context, in which two virtually uncorrelated dimensions are necessary to describe the political attitudes of the mass public.

The degree of alignment between economic and social attitudes may also depend on where on the left-right continuum the person falls. In the United States, as in other two-party systems, economic right-wing attitudes tend to be rather strongly aligned with social conservatism (Malka et al., 2017). However, among those on the right there are groups of people with very different political, psychological, moral, and demographic profiles (Ellis and Stimson, 2012; Weber and Federico, 2013; Feldman and Johnston, 2014; Davis et al., 2016). The heterogeneity of the right on social issues has in the United States been argued to be due to the mobilization of the religious right in the 1970s and President Reagan’s ability to appeal to both economically and socially conservative voters, attracting these previously separate factions to the Republican Party (Lienesch, 1982; Dionne, 1991; Micklethwait and Wooldridge, 2005). This type of historically contextual and particular explanation would imply that the heterogeneity of the right is specific to the United States context. However, there could be something more general about this pattern, and we will investigate to what extent it applies to the Finnish political elite.

### Psychological Underpinnings of the Holding of Political Attitudes

The present research also sought to contribute to the current renaissance on research on how certain types of ideas appeal to certain types of people. Basic psychological motivations and tendencies have often been argued to underlie ideological differences. Two of the most researched individual difference predictors of political attitudes are right-wing authoritarianism (RWA; Adorno et al., 1950) and social domination orientation (SDO; Sidanius and Pratto, 1999), both of which refer to the acceptance of various forms of inequality and domination. People scoring high in RWA believe in an inherently chaotic world where order and stability must be created by submitting to a strong authority. Although RWA was originally thought of as a more unitary construct (Adorno et al., 1950), subsequent personality scales designed to measure RWA have tended to assess three intercorrelated facets that together form RWA. The most popular of these scales, Altemeyer’s (Altemeyer, 1981, 1988) RWA scale, encompassed three distinct content areas, that is, Conventionalism, Authoritarian Submission, and Authoritarian Aggression. In this and similar conceptualizations of RWA (Duckitt et al., 2010; Mavor et al., 2010; Duckitt and Bizumic, 2013), Conventionalism (or Traditionalism) refers to the concern for maintaining or establishing normative consensus in groups and motivates attitudes such as acting in line with ingroup norms, Authoritarian Submission (Conservatism) refers to obeying people or institutions that promote norm compliance, and Authoritarian Aggression refers to punishing those who are breaking the norms of the ingroup. In the original formulation, however, authoritarianism was restricted to submissiveness and aggressiveness (Adorno et al., 1950, p. 228). Consistent with this formulation, Authoritarian Conventionalism has recently been argued to be best understood as a form of submissiveness to rules as well as to rulers (Smith and Hanley, 2018).

SDO was originally defined as the preference for group-based hierarchy and inequality. However, there is now mounting evidence that SDO needs to be conceptually divided into preference for systems where high-status groups dominate low-status groups (SDO Dominance) and preference for systems where inequality is maintained by hierarchy-enhancing ideologies and policies (SDO Anti-egalitarianism; Ho et al., 2015). Whereas SDO Dominance is related to blatant racism and other overtly aggressive intergroup phenomena, SDO Anti-egalitarianism has been defined as representing opposition to equality between groups, as supported by an interrelated network of subtle hierarchy-enhancing beliefs and social policies, and manifested in an affinity for ideologies and policies that maintain inequality, such as meritocracy.

Dual process models of political ideology argue that SDO and RWA constitute two distinct psychological motivations that underlie economic right-wing attitudes, and social conservatism, respectively (e.g., Duckitt et al., 2002; Duriez and Van Hiel, 2002; Duriez et al., 2005). Prior empirical research on the dual process model has focused on the mass public, and we sought to investigate whether a similar pattern can be found in the political elite. Some research suggests that the politically engaged may show higher consistency between their pre-political characteristics, such as their basic psychological motivations, and their political ideology, with the more knowledgeable being able to better select the ideology that satisfies their psychological needs (Federico and Goren, 2009; Federico et al., 2009; Malka et al., 2012). To what extent RWA and SDO underlie the political attitudes of political elites has not previously been investigated.

### The Purpose of the Present Research

Founding our study on the fundamental idea that there exist two separate dimensions of political attitudes – the economic and social dimensions (for a recent review and empirical support, see Malka et al., 2017) – we sought to investigate the extent to which these two dimensions are aligned among the almost 10,000 candidates in the Finnish 2017 municipal elections. Research conducted in the context of the United States two-party system suggests that the two dimensions are aligned among the political elite and that the alignment between the dimensions is stronger among those on the economic left and those more socially liberal, as compared to those on the economic right and more socially conservative. The novelty of our research lies in investigating these ideas in a multi-party context in which the correlation between the economic and social dimensions of political attitudes is virtually zero in the mass public (Malka et al., 2017). Our results could thus, first, help determine whether there among the political elite exists an association between the two dimensions of political attitudes even in contexts in which the mass public thinks of them as completely separate dimensions. Second, our results could help clarify whether there is some generalizability to the result that it is particularly those on the left and those socially liberal among whom the two dimensions are interrelated, or whether this pattern is specific to the United States. Third, we sought to investigate the possible psychological motivations underpinning political attitudes, or more precisely, whether aspects of RWA and SDO are associated with social conservatism and economic right-wing attitudes, respectively. The novelty of this research thus lies in the non-United States multi-party setting in which political attitudes are bi-dimensionally organized and in the focus on the political elite. Focusing on the attitudes of the political elite is by itself important – political elites often dominate the political environment, influence the attitudes and opinions of mass publics, and shape collective action (e.g., Statham and Geddes, 2006; Loizides, 2008); yet, there is very little large-scale survey research on the attitudes of the elites.

## Materials and Methods

### Participants and Procedure

Participants were those 9515 (4164 female; 48.8%) candidates in the Finnish 2017 municipal elections who had completed both the voting application hosted by the Finnish National Broadcasting company and the national newspaper Helsingin Sanomat. The former included items pertaining to authoritarian tendencies, and the latter included measures of right-wing economic attitudes and social conservatism. Candidates responded to the voting advice application before it was opened to the general public and were not aware of how other candidates had responded. The mean age of the candidates was 44.57 years (*SD* = 12.96; range 18–87). There were in total 36,616 candidates in the Finnish 2017 municipal elections. As compared to the 27,101 candidates who did not respond to the YLE and HS voting advice applications, those who had completed both of these voting applications (that is, our sample of 9515 candidates) were somewhat younger (the mean age of all candidates was 50 years) and women were somewhat overrepresented (40% of all candidates were women). All data is Open Data and available from the Helsingin Sanomat^1^ and YLE^2^ websites. Although the data is un-anonymous, local and national guidelines did not require ethics approval because all data is open access public data.

Highest education attained was coded into four categories, with categories representing participants with no secondary education (*n* = 222; 2.3%), with a vocational or high school degree (*n* = 992; 10.4%), with a bachelor’s degree or university of applied sciences degree (*n* = 2195; 23.1%), and participants with higher than bachelor’s degree (*n* = 5045; 53.0%). Altogether 1061 participants did not report their education.

Political engagement was coded into four categories, with categories representing no previous political experience (*n* = 3081; 32.4%), activity in political party at the municipal level (*n* = 3323; 34.9%), elected for a municipal office or council (*n* = 2929; 30.8%), and elected in national elections (*n* = 142; 1.5%). Forty participants (0.4%) did not respond to this question.

Yearly income was entered in six categories, with categories representing less than 20,000€ (*n* = 1418; 14.9%), 20,000–30,000€ (*n* = 1481; 15.6%), 30,000–50,000€ (*n* = 2689; 28.3%), 50,000–70,000€ (*n* = 979; 10.3%), 70,000–100,000€ (*n* = 384; 4.0%), and above 100,000€ (*n* = 148; 1.6%). A total of 2416 (25.4%) participants did not report their income. The average yearly income in Finland in the year 2015 was 28,750€, with around 40% earning below 20,000€, 20% earning 20,000–30,000€, 25% earning 30,000–50,000€, and the remaining 15% earning above 50,000€. The lowest income bracket was thus somewhat underrepresented in our sample.

In general, candidates were somewhat higher educated and had somewhat higher income than the population in general. However, the differences were relatively small, and the candidates do not constitute a socioeconomic elite. Municipal elections do not involve significant constitutional office and do not attract large scale media coverage. Only a small minority of candidates are “career politicians” who are politicians by occupation or have a history of active campaigning and public visibility.

### Measures

The items assessing economic and social attitudes that were used in the Helsingin Sanomat VAA were adapted from items in the World Values Survey and the European Social Survey (Ylä-Anttila, 2011). Economic attitudes were measured with four items: “Public services should be evermore privatized,” “If a situation arises in which it is necessary to either cut down on public services and social security benefits or raise taxes, raising taxes is the better option” (reverse scored), “Large income differences are acceptable, as an appropriate reward for differences of talent and dedication,” and “In the long run, the current levels of public service and social security benefits burden the public budget too much.” Each item was responded to on a scale from one (*completely disagree*) to five (*completely agree*), where higher numbers indicate more right-oriented responses. The mean score was 2.632 (*SD* = 1.012). Cronbach’s alpha internal consistency reliability was 0.800.

Social attitudes were measured with four items: “Gay and lesbian couples should have the same marriage and adoption rights as heterosexual couples” (reverse scored), “If the state offers to set up a refugee center in my home municipality, the offer should be accepted” (reverse scored), “School is too indulgent toward pupils. Stricter discipline would make schools better,” and “Traditional values, such as family values, religion, and patriotism, provide a sound basis for politics.” Each item was responded to on a scale from one (*completely disagree*) to five (*completely agree*). The higher the number, the more conservative the responses. The mean score was 2.718 (*SD* = 1.019) and alpha was 0.747.

The items that we employed to measure the psychological underpinnings of political attitudes were included in the VAA hosted by the Finnish national broadcasting company YLE. The length of the questionnaire had been set to thirty items, of which six were allocated to the measurement of psychologically relevant variables that could be expected to underlie political attitudes. Due to these space constrains set by YLE we could not use pre-existing scales, but were allowed to create, in close collaboration with journalists, six relatively simple, socially appropriate, and short items that would be interesting to those completing the measures and to readers, meaning that the items not be very similar to each other. These restrictions ruled out the possibility to use well-established multi-item scales. Instead, we tried to approach RWA and SDO – perhaps the two currently most popular individual difference predictors of political ideology – from various theoretical perspectives, with the intention of covering the breadth of the constructs rather than striving to maximize internal consistency reliability (this approach is often advocated in the development of short measures; e.g., Gosling et al., 2003; McCrae et al., 2011).

The possible precursors or psychological underpinnings of political attitudes were measured with six items that were responded to on a five-point scale ranging from -2 (*completely disagree*) to 2 (*completely agree*). The items were based on previous scales designed to assess the psychological underpinnings of political ideology, but we selected them and modified them to take into consideration the public nature of the responses. In essence, we sought to create items the responses to which would not be dictated too strongly by social desirability concerns (this meant excluding items assessing, e.g., preference for violent maintenance of oppressive hierarchies, blatant forms of dehumanization, racism).

The six items are presented in Table 1. Also presented are the item loadings from a three-factor solution that accounted for 66,099% of the total variance (maximum likelihood factor analysis with varimax rotation), with the three first factors explaining 29.606, 20.094, and 16.399% of the variance (the number of factors was determined by parallel analysis). The first factor was labeled SDO – Anti-egalitarianism. The subtle, hierarchy-enhancing beliefs (i.e., meritocratic beliefs) that load highly on this factor manifest an affinity to ideologies and policies that maintain inequality. The second factor, representing submissiveness to rules as well as to rulers, was labeled Authoritarian Conventionalism. The third factor, with high loading of the two items that emphasized reluctance to compromise and the need to fight, was labeled Authoritarian Aggression.

**Table 1:** Descriptives of items pertaining to SDO and RWA and their loadings on three factors.

Item	M (SD)	Items loadings		
		SDO Anti-egalitarianism	RWA conventionalism	RWA aggression
In Finland everyone has a fair shot at wealth and happiness.	-0.324 (1.275)	0.628	-0.104	0.122
If people were treated more equally we would have fewer problems in this country.	1.229 (1.036)	-0.516	-0.245	-0.104
Things were not better before. Our way of living has changed for the better.	0.731 (1.068)	0.071	-0.527	-0.172
It is more important for children to be curious and independent than well-behaved and obedient.	0.438 (1.152)	-0.131	-0.465	-0.061
What our country really needs is strong leadership that can solve problems without the need to compromise.	-0.534 (1.275)	0.039	0.115	0.498
All life is basically a competition for power and resources. To succeed one has to fight.	0.094 (1.236)	0.306	0.125	0.497

### Computation of Alignment Between Economic and Social Attitudes

Asendorpf (1990, 1992) showed that the overall Pearson correlation between two variables can be decomposed into the individual contributions of each of the pairs of scores from which a correlation is computed. In our case this means that the contribution of each individual candidate’s combination of economic and social attitudes to the overall correlation between economic and social attitudes could be computed. I.e., those individuals who occupy approximately the same rank on the scales of economic and social attitudes will contribute strongly to the overall correlation, whereas those individuals who occupy very different ranking positions contribute less, or negatively, to the overall correlation. The index that reflects each individual’s contribution, referred to as r_A_, is computed according to the Asendorpf (1992) formula: 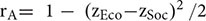, where z_Eco_ and z_Soc_ are z-scores for economic attitude and social attitude ratings standardized across the sample (this makes the index more robust to the non-normality of data). The mean of these r_A_ coefficients is equal to the Pearson product moment correlation between the two dimensions.

## Results

### The Relation Between Economic and Social Attitudes

**Figure 1:**
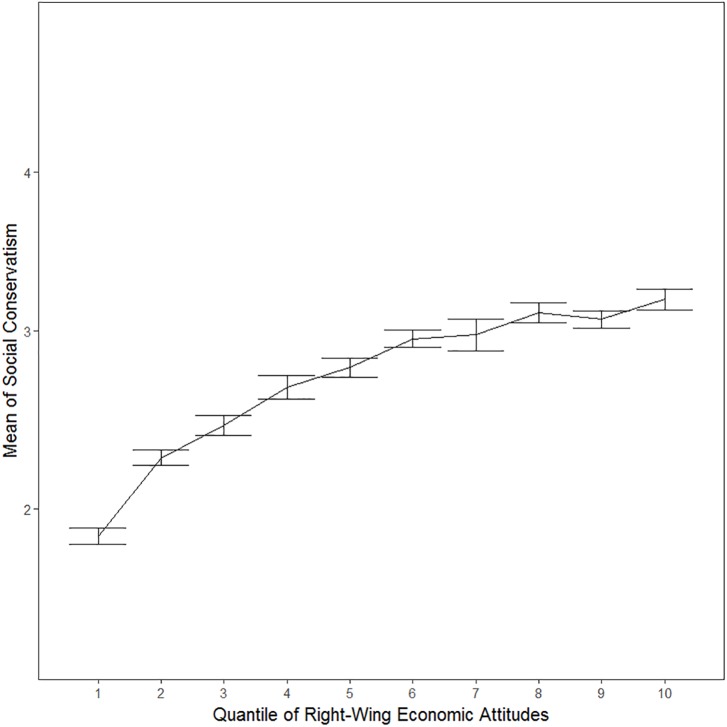
Social attitudes with 95% CIs as a function of deciles of economic attitudes.

**Figure 2:**
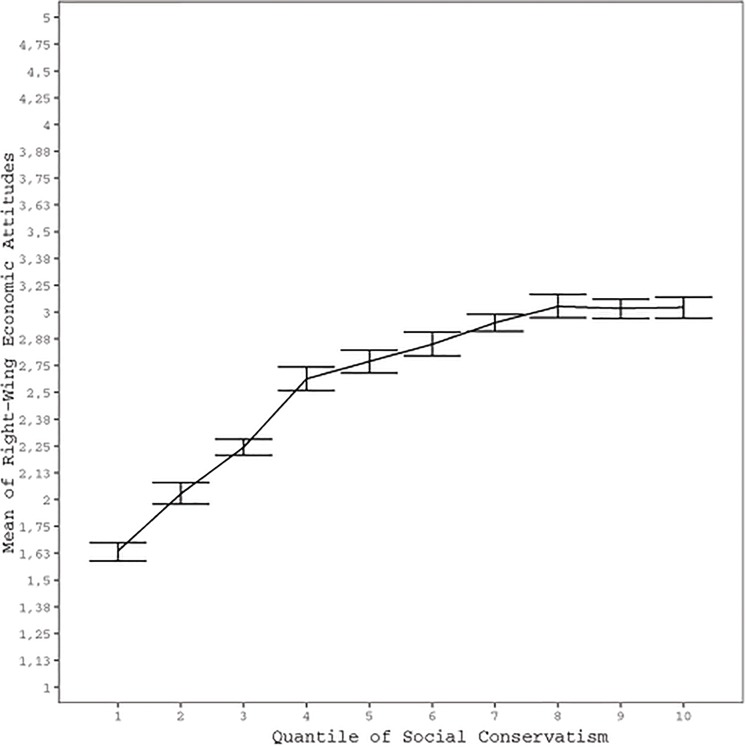
Economic attitudes with 95% CIs as a function of deciles of social attitudes.

To illustrate the association between economic and social attitudes, we plotted social attitudes as a function of economic attitudes after first dividing the sample into deciles according to the latter. Inspection of Figure 1 shows that those on the economic left also tend to be rather liberal in terms of social attitudes. By contrast, those on the right appear to be freer to choose their social attitudes – the mean scores of even the right-most deciles in terms of economic attitudes fall around the mid-point of the social conservatism scale. A similar pattern can be seen in Figure 2, which depicts economic attitudes as a function of social conservatism. The more liberal tend to be rather left-wing, but the socially conservative appear not to be similarly constrained in terms of their economic attitudes. Thus, being economically right-oriented does not require that one is also socially conservative, or vice versa. On the other hand, those on the economic left tend to be rather socially liberal, and those who are socially liberal tend to be rather left in terms of economic attitudes.

The correlations shown in Table 2 confirm what visual inspection of Figure 1, 2 already suggested. The correlation between right-wing economic attitudes and social conservatism was *r* = 0.410. This implies that the political elite, even in the Finnish context in which the mass public organizes their political attitudes on two independent dimensions, will tend to organize their political attitudes on two moderately aligned dimensions. Moreover, the correlation between these dimensions is more strongly driven by those on the left and those more liberal: correlations and partial correlations (age and sex removed) between the Asendorpf index and being on the right and being conservative ranged from around *r* = -0.17 to *r* = -0.20.

**Table 2:** Correlations and partial correlations between all variables.

	2.	3.	4.	5.	6.	7.	8.	9.	10.	11.
(1) Sex	0.050	-0.133	0.108	0.033	0.133	0.193	0.112	-0.017	0.139	-0.084
(2) Age		0.151	0.289	0.275	-0.009	0.133	-0.063	0.070	0.016	0.031
(3) Education			0.323	0.094	0.050	-0.132	0.022	-0.106	-0.122	0.051
(4) Income		0.311		0.246	0.230	0.028	0.168	-0.091	0.006	-0.015
(5) Experience		0.058	0.179		0.050	0.044	0.009	-0.030	-0.022	0.027
(6) Right-wing		-0.141	-0.036	0.001		0.410	0.593	0.040	0.299	-0.197
(7) Conservative		0.063	0.225	0.047	0.424		0.363	0.457	0.463	-0.177
(8) SDO Anti-egalitarianism		0.044	0.174	0.033	0.586	0.379		0.041	0.253	-0.112
(9) RWA conventionalism		-0.141	-0.117	-0.049	0.063	0.467	-0.049		0.261	-0.034
(10) RWA aggression		-0.116	-0.014	-0.038	0.295	0.467	0.261	0.274		-0.110
(11) r_A_		0.033	-0.022	0.016	-0.226	-0.185	-0.121	-0.043	-0.127	

*Numbers below the diagonal are partial correlations with age and sex partialled out. Sex was coded 0 = Female, 1 = Male. r_*A*_ is the Asendorpf index and indicates the extent to which economic and social attitudes are aligned. *N* varies between 9342 and 9515, except for correlations that involved Income (*n* varied around 7000) or Education (*n* varied around 8400). Correlations larger than 0.03, 0.04, and 0.05 are significant at *p* = 0.01, 0.001, and 0.0001, respectively*.

Besides suggesting, as expected, that it is particularly those on the left and those socially liberal among whom the two dimensions of political attitudes are interrelated, Figure 1, 2 further suggest that it is particularly those who are furthest to the left that are the most liberal. To investigate this, we explored the extent to which the association between the two dimensions is curvilinear. More specifically, we ran a regression analysis in which we predicted right-wing orientation with both conservatism and with conservatism squared, entered at steps 1 and 2 of the regression analysis, respectively. Adding conservatism squared to the regression analysis increased the amount of explained variance from adjusted *R*^2^ = 0.168 (Step 1) to adjusted *R*^2^ = 0.205 (Step 2). This change of 0.36 in adjusted *R*^2^ was statistically significant [*F* change (1,9512) = 435.275, *p* < 0.001] and both the linear (β = 1.425; *t* = 28.790) and quadratic (β = -1.033; *t* = 20.863) terms were statistically significant at step 2 of the regression analysis (both *p* < 0.001). Similarly, in the prediction of conservatism, adding right-wing orientation squared to right-wing orientation added the amount of explained variance from adjusted *R*^2^ = 0.168 (Step 1) to adjusted *R*^2^ = 0.192 (Step 2). This change of 0.024 in adjusted *R*^2^ was statistically significant [*F* change (1,9512) = 282.002, *p* < 0.001] and both the linear (β = 1.227; *t* = 24.778) and quadratic (β = -0.832; *t* = 16.793) terms were statistically significant at step 2 (both *p* < 0.001). These exploratory regression analyses confirmed the existence of the type of curvilinear quadratic effect that is suggested by Figure 1, 2; it appears it is particularly those that are very far to the left (liberal) that are the most socially liberal (left).

Concerning the psychological underpinnings of political attitudes, right-wing economic attitudes were strongly correlated with SDO Anti-egalitarianism (*r* = 0.593) and moderately correlated with Authoritarian Aggression (*r* = 0.299), but not at all with Authoritarian Conventionalism (*r* = 0.004). Social conservatism, on the other hand, was moderately correlated with both Authoritarian Conventionalism and Authoritarian Aggression (*r* = 0.457 and *r* = 0.463, respectively), but also with SDO Anti-egalitarianism (*r* = 0.363). This pattern of results generally conforms to the dual-process model of political attitudes, according to which SDO underlies right-wing economics attitudes and RWA socially conservative attitudes.

## Discussion

Arguably the most important finding of the present research was that even in multi-party system in which the masses organize their political attitudes on two independent dimensions, these two dimensions tend to be moderately correlated among the political elite, and that this alignment is driven by those on the (far) left and the socially (very) liberal. Another important finding was that the currently popular dual-process model of political ideology can help understand not only the political ideology of the masses, but also that of the political elite.

Our results suggest that the association between economic right-wing orientation and social conservatism, or, to even greater extent, between left-wing economic orientation and social liberalism, may be more intrinsic than is sometimes argued. More precisely, our results can be interpreted as challenging accounts that suggest these associations to be purely contextual results of particular historical processes (e.g., Lienesch, 1982; Dionne, 1991; Micklethwait and Wooldridge, 2005). Our results can also be interpreted as challenging accounts according to which other constellations, such as that between economic left-wing attitudes and social conservatism to be more coherent (e.g., Malka et al., 2017). Our results also suggest that although the political elite may in several ways be more rational in their decision-making than the mass public (e.g., less prone to certain judgment and decision-making biases; for a review, see Hafner-Burton et al., 2013), their political attitudes are in part based on underlying psychological motivations.

### The Relation Between Economic and Social Attitudes

The relatively strong correlation between economic right-wing orientation and social conservatism could be considered surprising – the space of West European politics has in recent decades become clearly two-dimensional (Bornschier, 2010) and the correlation between the two dimensions is virtually zero in more representative samples of Finns (Malka et al., 2017). Thus, even in a multi-party system and in a context in which the masses organize their political attitudes on separate and very distinct economic and social dimensions, the political elite organizes their economic and social attitudes more along a single dimension.

Our results on the relation between economic and social attitudes are consistent with results obtained in the United States, according to which alignment between the economic and social dimensions is conditional on a person’s level of political engagement – the cultural and economic attitudes of the more politically engaged are more strongly aligned (Sidanius and Duffy, 1988; Federico and Schneider, 2007). However, our results may require a different type of explanation than the one often referred to in the United States context. In the United States two-party system, the politically engaged have been argued to align their views to their party’s position on most if not all issues, as a consequence of which one dimension will suffice to describe individual differences in political attitudes. It is less clear why, in a multi-party Western European system such as Finland, the two dimensions would be more strongly aligned among the politically engaged. That the correlation between the two dimensions was driven by those on the left and those more liberal could, we believe, offer some clues for why these dimensions may often be correlated among the politically engaged. I.e., those on the economic left tend to have stronger equality concerns. A frequent reason for people to sympathize with the left is a perception of the world as unfair – inequality is perceived as illegitimate and unjustified (Jost and Hunyady, 2002; Jost et al., 2003). Moreover, those on the left are more likely to interpret inequalities as the result of structural factors such as discrimination, stereotyping, and exclusion from social networks (e.g., Gurin, 1985; Major and Schmader, 2001). This means that inequality in the economic sphere is intertwined with inequality in the social sphere. Forms of economic inequality, such as between management and labor, the rich and the poor, corporations and consumers, thus becomes interwoven with forms of social inequality, such as between white and black, men and women, migrants and natives. The dismantling of one type of inequality, be it economic or social, requires the dismantling of the other type of inequality.

The above line of reasoning implies that for left-wing people, concerns about economic injustice could be expected to motivate more liberal social attitudes; i.e., views according to which people should be treated similarly regardless of, for instance, religious affiliation, societal status, cultural background, ethnicity, sexual preferences, or gender. This could happen because left-wing people tend to value equality *per se*, because they tend to see social and economic injustices as intertwined (e.g., Gurin, 1985; Major and Schmader, 2001), or both. Researchers with data that includes measures of political attitudes and measures of moral concerns, such as measures based on moral foundations theory (Graham et al., 2011) or on Schwartz (1992); Schwartz et al. (2012) theory of human values, could more explicitly test the idea that equality concerns have a role to play in explaining the relation between economic and social attitudes.

An unexpected finding was that it is particularly those who were very far to the left (very liberal) that were liberal (to the left). This pattern could be driven by general response style – those who provided extreme responses to the items concerning economic attitudes may be more likely to do so also in the domain of social attitudes – or it could be a more substantive effect. Even in an elite sample, in which everyone can be presumed to be high in both political knowledge and engagement, those furthest to the economic left or those most socially liberal may be the most knowledgeable or most engaged, leading to stronger alignment between the two dimensions of political attitudes (Sidanius and Duffy, 1988; Federico and Schneider, 2007). Whether this effect replicates in other contexts and its causes should be interesting topics for future research.

### The Dual Model Process of Political Ideology

In the last decades, SDO and RWA have been among the constructs that have attracted the most research attention in terms of understanding the psychological underpinnings of political attitudes. The dual-process model suggests that SDO may underlie a preference for right-wing economic attitudes, and RWA a preference for socially conservative attitudes. Such findings have been reported in other Western European contexts (e.g., Duckitt et al., 2002; Duriez and Van Hiel, 2002; Duriez et al., 2005; Cizmar et al., 2014) and our results are largely consistent with these findings: SDO Anti-egalitarianism was strongly correlated with right-wing economic attitudes, and Authoritarian Conventionalism and Aggression were both moderately correlated with social conservatism. However, two further findings require comment.

SDO Anti-egalitarianism was not only strongly associated with right wing-economic views, but also moderately strongly associated with social conservatism. This could be considered surprising given that Finland is often considered a Nordic welfare state in which democracy has at least historically restrained the power of capital and income inequality is low. Anti-egalitarian people could thus be expected to yearn for change rather than for maintaining the social fabric. However, for almost three decades now, Finnish governments have pursued a neoliberal doctrine and pushed for the restructuring of the role of the state in a manner highly sympathetic toward the views of the business elite. The continuing rise in income inequality and the ongoing dismantling of the welfare state may predispose those with an anti-egalitarian bent to be rather happy with the current status quo.

The second finding that could be considered unexpected was that Authoritarian Aggression was associated not only with social conservatism, but also with right-wing economic views. It seems possible that people endorsing these rather aggressive views could, as formulated by sociologist Arlie Hochschild (2016), feel that while they work hard, others, usually immigrants and minorities, are cutting in line, which leads to anger and outrage toward a political system that is seen as favoring others. Such feelings could be expected to be especially accentuated in a well-fare state such as Finland that redistributes resources to the poor, and manifest in political rhetoric and policies that call for dismantling social welfare programs. To the best of our knowledge, Authoritarian Aggression has not in other samples been associated with economic right-orientation. However, in Sweden, also a welfare state, low regard for Fairness and Harm considerations was reported to be associated with opposition to equality and with placing oneself as right-wing on a scale that assessed political orientation (Nilsson and Erlandsson, 2015). This may not be true in other contexts. For instance, in a United States sample, a measure of dogmatic aggression toward those with other beliefs and values was correlated only with social conservatism, not right-wing economical orientation (Crowson, 2009). Nevertheless, the current results obtained from Finland and the previous results from Sweden together suggest that the more aggressive aspects of authoritarianism may in welfare states be associated with right-wing economic attitudes. This can be considered rather surprising, as neither the Finnish nor Swedish populist parties, both of which employ discourses that aim to protect the welfare state from the drain of immigration, tend to question the redistributive welfare state *per se* (Nordensvard and Ketola, 2015). Alleviating fears that undeserving groups, such as migrants, are taking advantage of the welfare state could help both curtail aggressiveness toward these groups and increase support for the welfare state. Particularly economic arguments for the necessity of immigration in upholding the welfare state could be effective in combatting authoritarian aggressiveness.

### Limitations and Conclusion

Perhaps the most obvious limitation of the present research was our reliance on only six items to measure various aspects of SDO and RWA. It would have been highly interesting to include also items that tapped into, for instance, SDO Dominance, but this was not possible due to the restrictions set by the VAA provider and also due to fear that social desirability concerns would drive responses to items with very blatant content.

Regarding generalizability, our sample consisted of candidates seeking political office. The sample must be considered biased in many ways. Willingness to join a political organization and run for public office expresses not only a high level of political engagement, but may also express, e.g., a greater need for prestige or power, a wish to help others, or a desire to be in the public eye. The demographic data suggest that our sample was somewhat better off than the general population, but the psychological differences may well be much more pronounced.

Despite the above limitations, our results allow us to conclude that (a) even in a context in which the structuring of political spaces among the masses is clearly two-dimensional (Malka et al., 2017), economic and social attitudes are more aligned among the political elite, (b) the moderate alignment between the two dimensions is due to those on the economical left being more socially liberal and vice versa, and (c) various aspects of SDO and RWA underlie right-wing economic attitudes and social conservatism, respectively. Recent reviews and meta-analyses of the field have suggested that the relations between underlying psychological dispositions and political attitudes vary considerably based on contextual factors (Federico and Malka, 2018). Our results are consistent with these ideas, lending support to the idea that results from research conducted with the mass public cannot be generalized to the political elite. Moreover, the associations between psychological constructs, such as SDO and RWA, and the holding of political attitudes may vary from context to context. Before any generalizations can be drawn, we need much more samples from diverse contexts. This is also true for our findings regarding the alignment between economic and social attitudes. Above we argue for a certain coherence between being economically left-wing and socially liberal – both being left and being liberal may be driven by underlying equality concerns. However, whether such an alignment between these dimensions can be found in other contexts, particularly non-Western contexts, needs to be investigated in future research.

## Author Contributions

J-EL ran the data analysis and drafted the manuscript. MK developed the study design and coordinated the study and participated in the drafting of the manuscript.

## Conflict of Interest Statement

The authors declare that the research was conducted in the absence of any commercial or financial relationships that could be construed as a potential conflict of interest.

## Footnotes

1 https://github.com/HS-Datadesk/avoindata/tree/master/vaalikoneet/kuntavaalit2017

2 https://yle.fi/uutiset/3-9526290
